# Development of the great recess framework – observational tool to measure contextual and behavioral components of elementary school recess

**DOI:** 10.1186/s12889-018-5295-y

**Published:** 2018-03-22

**Authors:** William V. Massey, Megan B. Stellino, Sean P. Mullen, Jennette Claassen, Megan Wilkison

**Affiliations:** 1 College of Public Health and Human Sciences, School of Biological and Population Health Sciences, Kinesiology Program, Oregon State University, Milam Hall 118L, 2520 SW Campus Way, Corvallis, OR 97331 USA; 2 School of Sport and Exercise Science, University of Northern Colorado, Greeley, CO 80639 USA; 3 Department of Kinesiology and Community Health, University of Illinois at Urbana-Champaign, Urbana, IL 61822 USA; 4 Playworks Education Energized, 380 Washington Street, Oakland, CA 84607 USA; 5 Department of Occupational Therapy, Concordia University Wisconsin, Mequon, WI 53097 USA

**Keywords:** Recess, Evaluation, School-based physical activity, Social environment

## Abstract

**Background:**

Physical activity (PA) remains the primary behavioral outcome associated with school recess, while many other potentially relevant indicators of recess remain unexamined. Few studies have assessed observations of teacher/student interactions, peer conflict, social interactions, or safety within the recess environment. Furthermore, a psychometrically-sound instrument does not exist to examine safety, resources, student engagement, adult engagement, pro-social/anti-social behavior, and student empowerment on the playground. The purpose of the current study was to develop a valid, and reliable, assessment tool intended for use in measurement of the contextual factors associated with recess.

**Methods:**

An iterative and multi-step process was used to develop a tool that measures safety and structure, adult engagement and supervision, student behaviors, and transitions at recess. Exploratory structural equation modeling (Mplus v. 7.4) was used to examine the underlying measurement model with observational data of the recess environment collected at 649 school-based recess periods that spanned across 22 urban/metropolitan areas in the USA. Data were also collected by two researchers at 162 recess sessions across 9 schools to examine reliability.

**Results:**

A 17-item observation instrument, the Great Recess Framework – Observational Tool (GRF-OT), was created. Findings of exploratory structural equation modeling (ESEM) analyses supported factorial validity for a 4-factor solution and linear regressions established convergent validity where ‘structure and safety’, ‘adult engagement and supervision’, and ‘student behaviors’ were all significantly related to observed activity levels. Each sub-scale of the GRF-OT showed adequate levels of inter-rater reliability and test-retest reliability analysis indicated a higher level of stability for the GRF-OT when using a three-day average across two time points as compared to a two-day average.

**Conclusions:**

Initial evidence for a valid, and reliable, assessment tool to observationally measure the recess environment with a specific focus on safety, resources, student engagement, adult engagement, pro-social/anti-social behavior, and student empowerment was established in this study. Use of the GRF-OT can inspire evaluation, and subsequent intervention, to strategically create consistent, appropriate, and engaging school recess that impact children’s physical, cognitive, social and emotional development.

**Electronic supplementary material:**

The online version of this article (10.1186/s12889-018-5295-y) contains supplementary material, which is available to authorized users.

## Background

Over the past decade, much attention has been paid to school-based recess and the implications of recess on child development. In 2013, the American Academy of Pediatrics released a policy statement citing the crucial role of recess within schools [1]. Authors of the policy statement presented evidence to suggest that recess provides cognitive and academic benefits, social and emotional benefits, and physical benefits to the child. More recently, the United States (U.S.) Centers for Disease Control (CDC) and SHAPE America [2] released evidence-based guidelines for recess strategies citing many of the same academic, social, emotional, and physical benefits recess can have on students. Within these guidelines, strategies to help facilitate positive outcomes included: (a) leadership decisions in which recess plans are developed, space is designated, and adults are properly trained; (b) communicating and enforcing behavioral and safety expectations such as communication and conflict resolution skills; (c) creating an environment that is supportive of physical activity by ensuring, among other things, that proper space and equipment is available; (d) engaging the school community to support recess; and (e) collecting data on the recess environment and potential outcomes that recess may affect (e.g., student and school outcomes). Thus, stakeholders have identified a high-quality recess as one that includes planning, structure, access to space and equipment, positive student behaviors, trained adult staff, and high levels of physical activity (PA). However, to date, there is a lack of assessment tools to measure the context of recess that might support positive child development beyond levels of physical activity.

In considering the possible benefits associated with recess, the majority of research findings, specifically within the public health domain, have focused on recess as instrumental to achieving PA related goals. Research findings within this domain have consistently shown that recess provides a needed contribution to children’s levels of PA during the school day [3–5]. Specifically, a RWJF report [5] stated that recess accounted for 42% of children’s opportunities to be physically active in school, while Erwin et al. [3], reported that recess accounted for up to 44% of step counts during the school day. These data should not be overlooked, as children spend approximately 40% of their waking hours at school [4] and 60% of school districts have no formal recess policy [6]. Moreover, only 22% of school districts in the U.S. require daily recess for elementary school students, with less than half of these requiring at least 20 min of recess per day. Given the PA benefits, recess has also been implicated as a potential contributor to children’s cognitive and academic development. This argument is predominantly made by those showing links between PA and health to cognitive and academic performance. For example, results from various studies and meta-analyses have highlighted that higher-fit children outperformed their lower-fit counterparts in laboratory measures of inhibition [7, 8], working memory [9], and overall cognitive functioning [10, 11]. Additional studies have also demonstrated body mass index and levels of inflammation are inversely related to various measures of cognitive performance [12, 13]. School-specific data have suggested that school-based PA interventions have the ability to positively affect academic performance [14, 15], with additional evidence to support a dose-response relationship [16]. Despite this, very limited evidence specific to increased PA through recess has shown a beneficial impact on academic well-being and classroom behavior [17, 18].

Aside from physical and cognitive benefits, it has been proposed that participation in play can help facilitate the development of social and emotional skills such as cooperative goal setting, teamwork, and emotional regulation [19]. Naturally, these ideas have been transposed into the recess environment. Proponents of these ideas have suggested that participation in physically active games during recess is positively associated with pro-social behaviors such as the ability to develop peer relationships, sharing, problem solving, and conflict resolution [20]. However, recent experimental data show a null effect on social skills competency following recess intervention programs [21, 22]. In considering prosocial behaviors and social skill development through recess, Left and colleagues [23] reported that the presence of organized games was predictive of higher levels of cooperative and intercultural play, and therefore might be an important mechanism for promoting social development through recess experiences. Thus, in considering the potential benefits of recess, the quality of the environment likely shapes how an individual experiences recess, and is likely to affect outcomes associated with participation in school-based recess.

In light of considering the contextual variables associated with recess, available data suggests that aside from potential benefits, recess is a place where violent and anti-social behavior can occur [24], and that some elementary school students view the playground as an unsafe space [25]. In a large survey of third through fifth grade students, Glew et al. [26] reported that the playground is the most likely place for bullying to occur at school, and that those involved (bullies, victims, bully-victims) had higher odds of feeling unsafe and sad at school. Moreover, victims and bully-victims had higher odds of low academic achievement and lack of belonging at school than bystanders. If the playground environment is one in which bullying and anti-social behaviors occur, it becomes difficult to make the claim that this environment inherently contributes to social, emotional, and cognitive health. Altogether, recess has been shown to promote PA, and PA has been linked to cognitive, social, emotional, and physical benefits; yet the context (environmental, social, etc) of recess likely plays a mediating role as to whether or not recess is beneficial to children’s health. Given this collective premise, and in line with recommendations from the CDC and SHAPE America noted above [2], a need exists to measure the recess environment beyond PA levels.

To date, PA remains the primary outcome associated with recess, while ignoring other indicators inherent in the strategies released by the CDC and SHAPE America [2]. There remains a specific need to better understand environmental factors associated with recess, how students interact and communicate during recess, how conflict is managed during recess, the overall safety of the environment, and the role adults play in this process. Limited studies have assessed observed behaviors such as teacher/student interactions and peer conflict [18], or social interactions at recess [27], however no validated instrument exists to examine the recess environment, student behavior, and levels of adult engagement. The most common observational instruments cited in the literature appear to be the system for observing children’s activity and relationships during play (SOCARP) [27] and the system for observing play and leisure activity in youth (SOPLAY) [28]. These tools, however, are reliant on time sampling techniques that observe one child at a time and likely miss out on larger environmental influences. Others have created observational and coding schemes specific to study aims [18, 21, 23], however these do not allow for scalable research efforts. The purpose of the current study was to develop a valid, and reliable, assessment tool intended for use in measurement of the contextual factors and behaviors associated with recess. Within this, there was a specific focus on safety, resources, student engagement, adult engagement, pro-social/anti-social behavior, and student empowerment.

## Methods

An iterative and multi-step approach was taken to meet the purpose of the current study. Prior to formal data collection, item development took place over an extended period of time using multiple expert working groups. This process resulted in Great Recess Framework -Observational Tool (GRF-OT) to measure the context in which recess takes place and the behaviors that manifest within this context. The measurement model of the GRF-OT was then tested, followed by reliability and stability testing of the tool. The methods and procedures used to accomplish these goals are described below.

### Item development

The GRF-OT was developed over several iterations with the use of expert working groups and field testing driven by Playworks Education Energized (www.playworks.org). During the first iteration, a national team of recess researchers and practitioners developed a series of indicators thought to support physical activity and positive social development during recess. These indicators were derived from previous research [20, 29] as well as decades of professional practice. This team included three Playworks program directors, two Playworks Pro Trainers, the Playworks National Evaluation Director and the National Director of Quality Programs, along with input from researchers working in this area see [20, 29]. The initial items were then field tested by Playworks^1^ program directors at recess sessions across the United States. A second working group of four different Playworks program directors, two different Playworks Pro Trainers, and the Playworks National Evaluation Director and the National Director of Quality was developed to improve these processes, as users felt the initial items were not user friendly and did not follow a logical pattern for observation. Based on this groups experiences working in the field, higher order domains of safety, engagement, and empowerment were identified, with items corresponding to each of these domains subsequently created by this team. These items were then placed on a 3-point scale and sent to an external researcher with publications in this area for critical review and modifications i.e., [29]. This scale was piloted prior to being sent to a second group of external researchers [WVM, MBS] for critical review and further modifications. At this point, the items on the GRF-OT were moved from a 3-point to a 4-point response scale. Items were also modified for operational definition clarity and ease of use. The results of these processes can be found in the 24-item version of the GRF-OT (see Additional file 1).

### Procedures

Recess is often defined, and implemented, differently across various sectors but generally refers to discretionary breaks children have during the school day. For the purposes of the current study, recess observations took place during scheduled recess breaks immediately before or after the lunch period, typically lasting 15–30 min in duration. Schools maintained variable schedules, with some schools sending groups of students outside all at once, while others rotated the sessions with different children and different supervisors (e.g., only first through third graders at recess one, followed by only fourth and fifth graders at recess two). Outcome assessors arrived to the outdoor playground approximately 15 min before the scheduled recess session to complete a walkthrough of the playground and take any notes about the built environment. Outcome assessors then observed the entire recess session, taking notes on each item throughout the process. In all cases, the recess environment was completely visible to the outcome assessor, and outcome assessors were trained to move throughout the playground in a discreet manner in an effort to observe patterns of interaction and behavior. Final scoring of each item was completed immediately after the recess session and took into account the aggregate patterns of behavior throughout the duration of the recess session.

#### Validity

To test the measurement validity of the GRF-OT, data were collected at 649 individual school-based recess periods during the fall of 2016. These recess sessions spanned 495 schools across 22 urban, or metropolitan, areas in the United States of America. A list of data collection locations are available upon request.

#### Reliability

Eight graduate students were recruited as outcome assessors. These outcome assessors had no prior experience with observational data collection of recess, facilitation of school-based recess, or teaching in an elementary school. Thus, outcome assessors were novices related to school-based recess observational assessment. Outcome assessors were introduced to the items on the GRF-OT, the operational definitions, and trained in the scoring procedures. Each item was discussed in a series of workshops that allowed the outcome assessors to ask clarifying questions regarding scoring procedures. After initial discussions of each GRF-OT item, outcome assessors were instructed to read through a GRF-OT scoring manual that was created by the lead investigator. This training manual included each GRF-OT item, operational definitions, and examples for each item, scoring criteria for each item, as well as corresponding photos and videos to enhance the training process. Pilot observations were then conducted, followed by debriefing sessions to ensure clarity in the scoring instructions. Pilot data were not used in any analyses.

Data were then collected by two independent outcome assessors, blinded to one another’s scores, at 162 recess sessions. To ensure blinding of scores, data were entered by an independent staff member uninvolved in data collection. The 162 recess sessions took place across 9 schools, and data were collected at each school over a two-week period. In total, first grade students were observed in 47 sessions, second grade students in 52 sessions, third grade students in 51 sessions, fourth grade students in 52 sessions, fifth grade students in 52 sessions, and sixth grade students in 23 sessions.

### Data analysis

#### Validity

To examine the measurement model of the GRF-OT, exploratory structural equation modeling (ESEM) was used in MPlus version 7.4 [30]. In consideration of alternative data analysis strategies, exploratory factor analysis (EFA) and confirmatory factor analysis (CFA) were ruled out as an appopriate choice as EFA structures are often not supported by subsequent CFA [31], and CFA does not inherently permit correlated variance structures (i.e., conceptual overlap between similar items). Given that CFA requires each item to load on only one factor, statisticians [32] have offered ESEM as a more flexible and realistic approach to utilise in model development. Moreoever, inter-related constructs are consistent with the authors’ a priori conceptualization of recess domains, as safety, engagement, and student empowerment at recess are theorized to be inter-related constructs. By using the ESEM method, items were free to cross-load on multiple factors, rotation of the factor matrix was possible, and the researchers were able to calculate goodness of fit statistics typically associated with CFA [33, 34]. Additionally, this procedure allows for step-wise evaluation of the GRF-OT using multiple statistical criteria.

Decisions about the most appropriate model were made using the Chi Square (χ^2^) statistic, the Root Mean Square Error of Approximation (RMSEA), the Comparative Fit Index (CFI), the Tucker Lewis Index (TLI), and the Standard Root Mean Square Residual (SRMR). Generally, χ^2^ should have a non-significant value (*p* > .05, indicating model-to-data fit). However, the meaningfulness of this “absolute criterion” has been greatly debated, as it is sensitive to sample size and model complexity. Psychometricians have therefore recommended the use of multiple fit indices to be included in model evaluation process. Values at or above .95 for CFI and TLI, and values < .08 for SRMR and RMSEA have be used to indicate acceptable model fit, whereas values ≥ .98 and < .06 are preferred, respectively [35, 36]. There are no gold standards in model evaluation, but a conservative stance was adopted at this early stage of model development.

#### Reliability

To examine the reliability of the GRF-OT, weighted Kappa scores were calculated to examine the relative consistency of scoring between outcome assessors on each individual item. Kappa scores ranging from 0.8 to 1.0 are considered excellent agreement; scores ranging from 0.6–0.79 are considered good agreement; scores ranging from 0.4–0.59 are considered moderate agreement; scores ranging from 0.2–0.39 are considered fair agreement, and scores below 0.2 are considered poor agreement. In addition to individual item reliability, the inter-rater reliability of each sub-scale identified in the ESEM analysis, as well as the total GRF-OT, was examined by calculating an intra-class correlation coefficient (ICC) using a 2-way random effects model.

In addition to this inter-rater reliability, the test-retest reliability of the GRF-OT was examined. Data were averaged across three recess sessions in one week, and compared to data averaged across three recess sessions for the same school, and same time period, the following week. An ICC using a 2-way random effects model was used to compare the three day average in week one against the three day average in week two. The procedure was then replicated by computing a two-day average in week one (day 1 and day 2) and comparing that to a two-day average from week two (day 4 and day 5). A minimal detectable change (MDC) was calculated for both the two-day and the three-day average. The MDC is a practically important data point that allows users to assess actual change in recess, as opposed to expectated variability. The MDC is thought the be the change needed to ensure the recess climate is different than a previous observation. The following formula was used to calculate the MDC at a 95% confidence rate:

$$ \mathrm{MDC}=1.96\ \mathrm{x}\ 1.414\ \mathrm{x}\ \left(\mathrm{SD}\ \mathrm{x}\kern0.5em \left[\mathrm{sq}\ \mathrm{root}\ \mathrm{of}\ 1-\mathrm{ICC}\right]\right) $$

## Results

### Validity

**Fig. 1:**
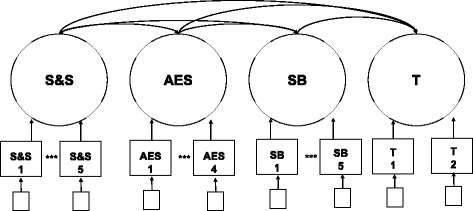
Measurement model of the GRF. S&S = Safety and Structure, AES = Adult Engagement and Supervision, SB = Student Behaviors, T = Transitions. CFI = .984, TLI = .971, RMSEA = .052, SRSM = .031

Prior to examination of the measurement validity of the GRF-OT, all data were screened to examine item frequencies and the distribution of scores. Four items were removed prior to conducting ESEM. Two items were removed as these were conditional items on the GRF-OT (see questions 16 and 18 in Additional file 1). An additional two items were removed as these items were insensitive to the range of scores (i.e., the items were given a score of “1” less than two times across the entire sample; see questions 3 and 11 in Additional file 1). The remaining items were retained and used in analysis. A preliminary analysis was conducted examining models that ranged from 2-factor to 6-factor solutions. This analysis suggested a four-factor model was most suitable for the data. After examining the individual item loadings on each factor, items 6, 12, and 13 had not loaded on any specific latent variable and were subsequently removed from factor analysis. An additional 2-through-6-factor solution ESEM analysis was then conducted which again suggested a 4-factor model as a suitable fit for the data (χ^2 =^ 202.02, *p* < .001; CFI = .984; TLI = .971; RMSEA = .052; SRSM = .031). Again, an individual item analysis was conducted and one item (item 9) had failed to load on any specific latent variable. Subsequent analyses indicated a similar model fit for a 4-factor solution (χ^2 =^ 188.72, *p* < .001; CFI = .984; TLI = .969; RMSEA = .056; SRSM = .031; Fig. 1). Given the importance given to levels of physical activity during recess, and that distinctness of item 13 in measuring physical activity, this item was retained to be included in the final observational tool. The final version of the observational tool can be found in Table 1. Factor loadings for individual items can be found in Table 2.

**Table 1:** Final GRF-OT Scale Items

Score	1	2	3	4
Safety and Structure 1	The play space for recess is unsafe due to hazards not identified as “no play” zones. There are significant safety concerns in almost all of the play spaces	The play space for recess has safety concerns due to hazardous areas on the majority of the playground not identified as “no play” zones	The play space for recess has some safety concerns. There are a few hazardous areas not identified as “no play” zones	The play space for recess has no safety concerns. It is clearly free of hazards and/or all unsafe areas are identified as “no play” zones
Safety and Structure 2	The play space for recess has no clearly identified boundaries for games (no cones, chalk, paint)	The play space for recess has a few boundaries identified but a large percentage of the play space does not have any game space marked	The play space for recess has many boundaries identified but a small portion of the play space does not have any game space marked	The play space for recess is well marked (cones, chalk, paint) and all game boundaries are clear
Safety and Structure 3	No fixed or unfixed recess equipment is available	Only fixed recess equipment is available OR only non-fixed recess equipment is available	Fixed recess equipment is available and there are limited amounts of non-fixed equipment	Fixed and non-fixed recess equipment is available to support multiple games and activities
Safety and Structure 4	Hardly any organized games and/or activities are available during recess	A limited number of organized games and/or activities are available during recess but there is limited variety	A limited number of organized games and/or activities are available during recess, but there is variety	A variety of organized games and/or activities are available during recess
Safety and Structure 5	Hardly any of the equipment provided is being used as intended and in a safe manner	Some of the equipment provided is being used appropriately but there are many instances of inappropriate use	Most of the equipment provided is being used appropriately but there are a few instances of inappropriate use	Almost all of the equipment provided is being used as intended and in a safe manner
Adult Engagement and Supervision 1	The adult to student ratio is more than 75:1	The adult to student ratio is between 51 and 74:1	The adult to student ratio is approximately 35–50:1	The adult to student ratio is less than 35:1
Adult Engagement and Supervision 2	Hardly any adults model positive culture (e.g. positive language, getting students involved, supporting conflict resolution skills, etc.)	A few adults model positive culture (e.g. positive language, getting students involved, supporting conflict resolution skills, etc.)	Many adults model positive culture (e.g. positive language, getting students involved, supporting conflict resolution skills, etc.)	Almost all adults model positive culture (e.g. positive language, getting students involved, supporting conflict resolution skills, etc.)
Adult Engagement and Supervision 3	Hardly any of the supervising adults are strategically positioned to view students in the recess play space (i.e., adults are all huddled together)	Some of the supervising adults are strategically positioned to view students in the recess play space, but many students are unsupervised	Many of the supervising adults are strategically positioned to view students in the recess play space, but some students are unsupervised	Almost all of the supervising adults are strategically positioned to view students in the recess play space
Adult Engagement and Supervision 4	Hardly any adults are playing games or engaged with students	A few adults are playing games and/or are engaged with students	Some adults are playing games and/or are engaged with students	Almost all adults are playing games and engaged with students
Student Behaviors 1	Hardly any games are initiated by students	A few games are initiated by students	Some games are initiated by students	Almost all games are initiated by students
Student Behaviors 2	There were several physical altercations between students	There were some physical altercations between students	There were few physical altercations between students	There were no physical altercations between students
Student Behaviors 3	Hardly any communication (verbal or nonverbal) between students is positive and encouraging towards each other	Very little communication (verbal or nonverbal) between students is positive and encouraging towards each other	Most of the communication (verbal or nonverbal) between students is positive and is encouraging towards each other	Almost all of communication (verbal or nonverbal) between students is positive and encouraging towards each other
Student Behaviors 4	There were several disagreements about rules between students that were disruptive to play	There were some disagreements about rules between students that were disruptive to play	There were few disagreements about rules between students that were disruptive to play	There were no disagreements about rules between students that were disruptive to play
Student Behaviors 5	Students demonstrate hardly any strategies for resolving conflicts on their own	Students demonstrate a few strategies for resolving conflicts on their own, but a lot of adult support was needed	Students demonstrate adequate strategies for resolving conflicts on their own, but some adult support was needed	Students demonstrate strategies to resolve their conflict without adult intervention or there was no evident conflict on the playground
Transitions 1	Hardly any transitions to recess from classroom are organized and smooth	Few transitions to recess from classroom are organized and smooth	Most transitions to recess from classroom are organized and smooth	All transitions to recess from classroom are organized and smooth
Transitions 2	Hardly any transitions to the classroom from recess are organized and smooth	Some transitions to the classroom from recess are organized and smooth	Most transitions to the classroom from recess are organized and smooth	All transitions to the classroom from recess are organized and smooth
Physical Activity	Hardly any students are involved in physically active play	Few students are involved in physically active play	Some students are involved in physically active play	Almost all students are involved in physically active play

**Table 2:** Individual item factor loadings and inter-rater reliability coefficients

Subscale/Item	Item Factor Loadings	Inter-rater Reliability
Safety and Structure 1	.505	% Agreement = 90.1
Safety and Structure 2	.554	*K(w)* = 0.691
Safety and Structure 3	.606	*K(w)* = 0.896
Safety and Structure 4	.741	*K(w)* = 0.791
Safety and Structure 5	.457	*K(w)* = 0.507
Adult Engagement and Supervision 1	.408	*K(w)* = 1.00
Adult Engagement and Supervision 2	.682	*K(w)* = 0.637
Adult Engagement and Supervision 3	.470	*K(w)* = 0.735
Adult Engagement and Supervision 4	.936	*K(w)* = 0.539
Student Behaviors 1	.504	*K(w)* = 0.782
Student Behaviors 2	.626	*K(w)* = 0.722
Student Behaviors 3	.797	*K(w)* = 0.846
Student Behaviors 4	.687	*K(w)* = 0.492
Student Behaviors 5	.815	*K(w)* = 0.709
Transitions 1	.708	*K(w)* = 0.760
Transitions 2	.663	*K(w)* = 0.689
Physical Activity Levels		*K(w)* = .538

Following the results of the ESEM, a linear regression was calculated in which each of the four sub-scales were entered as independent variables, and original item 13 (engagement in physical activity and play) was entered as the dependent variable. The overall model was significant (*p* < .001). Convergent validity was supported, as structure and safety (*p* < .001, β = .272), adult engagement and supervision (*p* < .001, β = .246), and student behaviors (*p* = .024, β = .102) were all significantly related to observed activity levels.

### Reliability

Results for inter-rater reliability of each item can be found in Table 2. One item (Item 1) lacked variability across the sample, thus a weighted Kappa score was unable to be calculated. The lack of variability is likely due to environmental consistencies, as all data were collected within the same school district. In lieu of a weighted Kappa score for this item, absolute percent agreement is reported. In addition to individual item reliability, each sub-scale of the GRF-OT showed adequate levels of inter-rater reliability. The structure and safety sub-scale of the GRF-OT contained five items with an ICC (2,1) for inter-rater reliability of 0.892 (95% CI, 0.856, 0.940). The adult engagement and supervision sub-scale of the GRF-OT contained four items with an ICC (2,1) for inter-rater reliability of 0.872 (95% CI, 0.830, 0.905). The student behaviour sub-scale of the GRF-OT contained five items with an ICC (2,1) for inter-rater reliability of 0.930 (95% CI, 0.903, 0.949). The transitions sub-scale of the GRF-OT contained two items with an ICC (2,1) for inter-rater reliability of 0.837 (95% CI, 0.784, 0.878). Inter-rater reliability on the total scale showed strong levels of agreement with an ICC (2,1) of 0.951 (95% CI, 0.932, 0.964).

Results of the test-retest reliability analysis indicated a higher level of stability for the GRF-OT when using a three day average (ICC = .949, 95% CI, .882, .979) across two time points as compared to a two day average (ICC = .855, 95% CI, .710, .930). Notably, the MDC for a three day average was calculated at 4.62, while only using a two day averaged yielded an MDC of 7.79. Thus, researchers planning to use the GRF-OT to measure improvement of recess over time should consider a three-day average score as sufficient to reduce the variability seen across daily recess sessions.

## Discussion

The purpose of the current study was to develop a valid, and reliable, assessment tool intended for use in measurement of the contextual factors associated with recess, and the behaviors that manifest within this context. Within this, were was a specific focus on safety, resources, student engagement, adult engagement, pro-social/anti-social behavior, and student empowerment. An evidence-informed, and psychometrically-sound, tool termed The Great Recess Framework – Observational Tool (GRF-OT) resulted from the data collection and analyses described in this paper.

Item development for the GRF-OT resulted in 17 items that each describe in short detail critical aspects of the recess environment with the particular area of focus noted above. Furthermore, we have provided reliability data for all initial items in the event school districts, practitioners, or evaluators find these items as relevant to their own evaluation efforts. Response formats for all items were according to a 1 (low quality) to 4 (high quality) rating of the particular focus and included succinct and distinct descriptions to anchor each possible score on the specific item. Subsequent thorough data collection and analyses revealed a four-factor measurement model and established measurement validity for the subscales including (1) structure and safety; (2) adult engagement and supervision; (3) student behaviors; and (4) transitions. Convergent validity was demonstrated in the significant associations of observed activity levels and the subscales of ‘structure and safety’, ‘adult engagement and supervision’, and ‘student behaviors’. Adequate levels of inter-rater reliability were found for all items, each of the four subscales and the entire GRF-OT. These findings create evidence of strong levels of consistency and agreement among raters across all items, scales and for the total tool. Last, examination of the stability of the GRF-OT revealed results that suggest three-day average of the scores indicate higher levels than two-day averages. Ultimately, these findings indicate that evaluation of a minimum of three days of recess best describe, in terms of stability, the features a specific recess environment.

Previous research shows school-based recess provides significant opportunity for, and accrual of, PA among children; thus contributing to physical health benefits [3–5]. Given this, it is not surprising that evaluations of recess have relied on examining self-reported, observed, or objective PA levels as a proxy measure for recess quality. Notably, to date, ‘success’ at recess has been a measure of how moderate-to-vigorously active children are during this time period, with the assumption that this level of activity can yield physical, cognitive, social, and emotional health benefits beyond the playground. Yet, if children are active in an unsafe environment that is prone to bullying, fighting, and lack of adult engagement, it would be naïve to assume this time period contributes to the holistic development of children, as recent policy articles might suggest [2]. A more holistic form of evaluation of the recess environment is therefore warranted. Furthermore, the acknowledgment that recess has implications for social, emotional, and cognitive health [2], should prompt the need for evaluation that considers contextual factors, student behaviors, and adult interactions during recess. Previous research has shown that access to equipment [37], levels of cooperative play [23], adult engagement and interactions [18], and conflict resolution skills [20, 29] are important to promoting a quality recess. While currently available assessment tools that examine child-level interactions are limited by time sampling methods that hone in on specifically targeted children, the GRF-OT provides researchers and practitioners with an opportunity to evaluate the overall environment and the nature of interactions that take place within it. As school-based recess remains at the forefront of policy discussions and decisions, there is a need to consider the quality of this environment for children in schools. The initiative forwarded by the CDC and SHAPE America [2] to develop evidence-based strategies for recess in schools should be assessed in a consistent manner inclusive of observed behaviors such as teacher/student interactions, peer conflict and safety at recess. The development of the GRF-OT is an important step in filling this evaluation gap currently identified within the extant literature.

### Limitations and directions for future research

The current study established the initial evidence for a valid and reliable assessment tool to measure the recess environment with a specific focus on safety and structure, adult supervision and engagement, student behaviors (communication, inter-personal interactions, conflict resolutions), and transitions to and from the recess environment. Despite its strengths, the current study is also not without limitations. First, it is important to note that the current evaluation framework represents an adult view of recess quality, which could likely differ from that of a child. Researchers in the field of public health have been examining the utility or instrumentality of children’s play, whereas children often view play as an end in and of itself [38]. Furthermore, item SB 4 gives a lower score when arguments arise around rules and game play, a process that might be a healthy part of negotiating play for children. Thus, future researcher may consider building on children’s perspectives of what is important for children during recess [39]. Second, to support a national data collection in examination of the measurement model of the GRF-OT, the research team partnered with Playworks to collect data from a wider range of schools and sources. As such, a majority of the validity data came from recess periods with a formal recess program, or intervention, in place. Additionally, the reliability data used in the current study was limited to one geographical region –a large urban public school district. Thus, there could have been environmental nuances, as well as policies and procedures that were germane to this region, but may differ when data is collected in various rural or other metropolitan areas. Specific to reliability, four items produced only moderate levels of agreement (SS 5, AES 4, SB 4, physical activity levels) prompting a need to more clearly define these items in training observers for data collection. The current study also does not address the inter-rater reliability between expert and novice users, a concern that needs to be addressed in future research. Finally, there is a need to establish construct validity of the GRF-OT using independent measures associated with recess. Researchers should examine relationships between the GRF-OT and objective levels of PA at recess, levels of student engagement at recess, and perceptions of student and adult safety at recess to further validate the existing tool.

## Conclusions

As efforts to understand the impact of school-based recess move beyond a focus on the PA-related aspects, children’s social, and emotional, development have emerged as relevant outcomes. Findings from extant research consistently suggest that the recess environment is conducive to facilitating positive growth in children’s emotional control, teamwork, cooperation, goal-setting, peer relationships, sharing, problem solving and conflict resolution [19, 20]. Yet for the benefits of recess to be fully realized contextual variables, student behavior, and adult interactions must be considered alongside PA in the evaluation of future research.

## Additional file

Additional file 1: Original GRF-OT. This file contains the original items used for testing the factorial validity of the GRF-OT. (DOCX 31 kb)

## Abbreviations

CDC: Centers for disease control
CFA: Confirmatory factory analysis
CFI: Comparative fit index
EFA: Exploratory factor analysis
ESEM: Exploratory structural equation modeling
GRF-OT: Great recess framework observational tool
ICC: Intra-class correlation
PA: Physical activity
RMSEA: Root mean square error of approximation
SRMR: Standard root mean square residual
TLI: Tucker lewis index

## Footnotes

1 Playworks is a U.S. based non-profit organization that seeks to provide safe and healthy play at school to children across the country. They currently reach nearly 1 million children through training and programming, with a goal providing safe and health play for 3.5 million children by 2020. For more information see www.playworks.org.

## References

[1] Murray R, Ramstetter C. The crucial role of recess in school. Pediatrics. 2012; 10.1542/peds.2012-2993. 10.1542/peds.2012-2993 23277311

[2] Centers for Disease Control, SHAPE America. Recess. In: Physical activity. U.S. Department of Health & Human Services. 2017. https://www.cdc.gov/healthyschools/physicalactivity/recess.htm. Accessed 26 Apr 2017.

[3] Erwin H, Abel M, Beagle A, Noland MP, Worley B, Riggs R (2012). The contribution of recess to children's school-day physical activity. J Phys Act Health.

[4] Gao Z, Chen S, Stodden DF. A comparison of children's physical activity levels in physical education, recess, and exergaming. J Phys Act Health. 2015; 10.1123/jpah.2013-0392. 10.1123/jpah.2013-0392 24828561

[5] Robert Wood Johnson Foundation. Recess rules: Why the undervalued playtime may be America’s best investment for healthy kids and healthy schools report. In: Research. Robert Wood Johnson Foundation. 2007. http://www.rwjf.org/content/dam/farm/reports/reports/2007/rwjf18060. Accessed 24 June 2014.

[6] Centers for Disease Control and Prevention, Bridging the Gap Research Program. Strategies for supporting recess in elementary schools, update for the 2012–13 school year. In: Nutrition, Physical Activity, & Obesity. U.S. Department of Health and Human Services. 2014. https://www.cdc.gov/healthyschools/npao/pdf/LWP_Recess_Brief_2012_13.pdf. Accessed 26 Apr 2017.

[7] Pontifex MB, Raine LB, Johnson CR, Chaddock L, Voss MW, Cohen NJ, et al. Cardiorespiratory fitness and the flexible modulation of cognitive control in preadolescent children. *J Cogn Neurosci.* 2011; 10.1162/jocn.2010.21528. 10.1162/jocn.2010.21528 20521857

[8] Wu CT, Pontifex MB, Raine LB, Chaddock L, Voss MW, Kramer AF, et al. Aerobic fitness and response variability in preadolescent children performing a cognitive control task. Neuropsychology. 2011; 10.1037/a0022167. 10.1037/a0022167 PMC3086950 21443340

[9] Chaddock L, Pontifex MB, Hillman CH, Kramer AF. A review of the relation of aerobic fitness and physical activity to brain structure and function in children. J Int Neuropsychol Soc. 2011; 10.1017/S1355617711000567. 10.1017/S1355617711000567 22040896

[10] Hillman CH, Erickson KI, Kramer AF. Be smart, exercise your heart: Exercise effects on brain and cognition. Nat Rev Neurosci. 2008; 10.1038/nrn2298. 10.1038/nrn2298 18094706

[11] Voss MW, Chaddock L, Kim JS, Vanpatter M, Pontifex MB, Raine LB, et al. Aerobic fitness is associated with greater efficiency of the network underlying cognitive control in preadolescent children. Neuroscience. 2011; 10.1016/j.neuroscience.2011.10.009. 10.1016/j.neuroscience.2011.10.009 PMC3237764 22027235

[12] Esteban-Cornejo I, Martinez-Gomez D, Garcia-Cervantes L, Ortega FB, Delgado-Alfonso A, Castro-Piñero J, et al. Objectively measured physical activity during physical education and school recess and their associations with academic performance in youth: The UP&DOWN study. J Phys Act Health. 2017; 10.1123/jpah.2016-0192. 10.1123/jpah.2016-0192 28032803

[13] Kamijo K, Khan NA, Pontifex MB, Scudder MR, Drollette ES, Raine LB, et al. The relation of adiposity to cognitive control and scholastic achievement in preadolescent children. Obesity (Silver Spring). 2012; 10.1038/oby.2012.112. 10.1038/oby.2012.112 PMC3414677 22546743

[14] DuBose KD, Eisenmann JC, Donnelly JE (2007). Aerobic fitness attenuates the metabolic syndrome score in normal-weight, at-risk-for-overweight, and overweight children. Pediatrics.

[15] DuBose KD, Mayo MS, Gibson CA, Green JL, Hill JO, Jacobsen DJ (2008). Physical activity across the curriculum (PAAC): rationale and design. Contemporary clinical trials.

[16] Davis CL, Tomporowski PD, McDowell JE, Austin BP, Miller PH, Yanasak NE, et al. Exercise improves executive function and achievement and alters brain activation in overweight children: A randomized, controlled trial. Health Psychol. 2011; 10.1037/a0021766. 10.1037/a0021766 PMC3057917 21299297

[17] Centers for Disease Control and Prevention. The association between school-based physical activity, including physical education, and academic performance. In: Heatlh & academics. U.S. Department of Health and Human Services. 2010. https://www.cdc.gov/healthyyouth/health_and_academics/pdf/pa-pe_paper.pdf. Accessed 26 Apr 2017.

[18] Massey WV, Stellino MB, Godbersen T, Holliday M, Rodia R, Kucher G, et al. The impact of a multi-component physical activity programme in low-income elementary schools. Health Educ J*.* 2017; 10.1177/0017896917700681.

[19] Miyamoto K, Huerta MC, Kubacka K. Fostering social and emotional skills for well-being and social progress. Eur J Educ. 2015; 10.1111/ejed.12118.

[20] Fortson J, James-Burdumy S, Bleeker M, Beyler N, London RA, Westrich L, et al. Impact and implementation findings from an experimental evaluation of Playworks: Effects on school climate, academic learning, student social skills, and behavior*.* In: RWJF Program Evaluations. Robert Wood Johnson Foundation. 2013. http://www.rwjf.org/content/dam/farm/reports/evaluations/2013/rwjf405971. Accessed 26 Apr 2017.

[21] Bunda A, Engelen L, Wyver S, Tranter P, Ragen J, Bauman A (2017). Sydney Playground Project: A cluster-randomized trial to increase physical activity, play, and social skills. J Sch Health.

[22] Mayfield CA, Child S, Weaver RG, Zarrett N, Beets MW, Moore JB. Effectiiness of a playground intervention for antisocial, proscoial, and physical activity behaviors. J Sch Health. 2017; doi:10.11/josh.12506. 10.1111/josh.12506 28382669

[23] Leff SS, Costigan T, Power TJ (2003). Using participatory research to develop a playground-based prevention program. J Sch Psychol.

[24] Anderson-Butcher D, Newsome W, Nay S (2003). Social skills intervention during elementary school recess: A visual analysis. Children Sch.

[25] Astor RA, Meyer HA, Pitner RO (2001). Elementary and middle school students’ perceptions of violence-prone school subcontexts. Elem Sch J.

[26] Glew GM, Fan MY, Katon W, Rivara FP, Bullying KMA (2005). psychosocial adjustment, and academic performance in elementary school. Arch Pediatr Adolesc Med.

[27] Ridgers ND, Stratton G, TL MK (2010). Reliability and validity of the System for Observing Children's Activity and Relationships during Play (SOCARP). J Phys Act Health.

[28] McIver KL, Brown WH, Pfeiffer KA, Dowda M, Pate RR. Development and testing of the Observational System for Recording Physical Activity in Children: Elementary School. R Q Exerc Sport. 2016; doi:10.1080*02701367.2015.1125994. 10.1080/02701367.2015.1125994 PMC4762057 26889587

[29] London RA, Castrechini S, Stokes-Guinan K (2013). Playworks implementation in 17 schools nationwide. Report submitted to the Robert Wood Johnson Foundation.

[30] Muthén L, Muthén B (2015). Mplus user’s guide [Computer software and manual].

[31] Marsh HW, Muthén B, Asparouhov T, Lüdtke O, Robitzsch A, Morin AJS, et al. Exploratory structural equation modeling, integrating CFA and EFA: Applications to students’ evaluations of university teaching. Struct Equ Modeling*.* 2009; 10.1080/10705510903008220.

[32] Marsh HW, Nagengast B, Morin AJS. Measurement invariance of big-five factors over the life span: ESEM tests of gender, age, plasticity, maturity, and La Dolce Vita effects. Dev Psychol. 2013; 10.1037/a0026913. 10.1037/a0026913 22250996

[33] Asparouhov T, Muthén B. Exploratory structural equation modeling. Struct Equ Modeling. 2009; 10.1080/10705510903008204.

[34] Marsh HW, Lüdtke O, Muthén B, Asparouhov T, Morin AJS, Trautwein U, et al. A new look at the big five factor structure through exploratory structural equation modeling. Psychol Assess. 2010; 10.1037/a0019227. 10.1037/a0019227 20822261

[35] Hu L, Bentler PM. Cutoff criteria for fit indexes in covariance structure analysis: Conventional criteria versus new alternatives. Struct Equ Modeling. 1999; 10.1080/10705519909540118.

[36] Marsh HW, Hau KT, Wen Z. In search of golden rules: Comment on hypothesis-testing approaches to setting cutoff values for fit indexes and dangers in overgeneralizing Hu and Bentler. Struct Equ Modeling. 2004; 10.1207/s15328007sem11032.

[37] Verstraete SJ, Cardon GM, De Clercq DL, De Bourdeaudhuij IM. Increasing children’s physical activity levels during recess periods in elementary schools: The effects of providing game equipment. Eur J Public Health. 2006; 10.1093/eurpub/ckl008. 10.1093/eurpub/ckl008 16431866

[38] Alexander SA, Frohlich KL, Fusco C. Problematizing “Play-for-Health” discourses through children’s photo-elicited narratives. Qual Health Res. 2014; 10.1177/1049732314546753. 10.1177/1049732314546753 25147214

[39] Pawlowski SK, Schipperijn J, Tjornhoj-Thomsen T, Troelsen J. Giving children voice: Exploring qualitative perspectives on factors influencing recess physical activity. European Physical Education Review. 2016; 10.1177/1356336X16664748.

