# Supplementary material for: Development of the great recess framework – observational tool to measure contextual and behavioral components of elementary school recess
Source: BMC Public Health. 2018 Mar 22;18:394. doi: 10.1186/s12889-018-5295-y (PMC5863843; doi:10.1186/s12889-018-5295-y)
Supplement: Supplementary file 1 — Original GRF-OT. This file contains the original items used for testing the factorial validity of the GRF-OT. (DOCX 31 kb) [file 12889_2018_5295_MOESM1_ESM.docx]

| **1** | | **2** | **3** | **4** | **Kappa (w)** |
| --- | --- | --- | --- | --- | --- |
| 1 | The play space for recess is unsafe due to hazards not identified as "no play" zones. There are significant safety concerns in almost all of the play spaces | The play space for recess has safety concerns due to hazardous areas on the majority of the playground not identified as "no play" zones | The play space for recess has some safety concerns. There are a few hazardous areas not identified as "no play" zones | The play space for recess has no safety concerns. It is clearly free of hazards and/or all unsafe areas are identified as "no play" zones | 90.1%  Agreement |
| 2 | The play space for recess has no clearly identified boundaries for games (no cones, chalk, paint) | The play space for recess has a few boundaries identified but a large percentage of the play space does not have any game space marked | The play space for recess has many boundaries identified but a small portion of the play space does not have any game space marked | The play space for recess is well marked (cones, chalk, paint) and all game boundaries are clear | .691 |
| 3 | The play space for recess has many immediate safety concerns due to the size and location | The play space for recess has some immediate safety concerns due to the size and/or location | The play space for recess has very few immediate safety concerns due to the size or location. | The play space for recess is appropriate in that there are no immediate safety concerns | .619 |
| 4 | No fixed or unfixed recess equipment is available | Only fixed recess equipment is available OR only non-fixed recess equipment is available | Fixed recess equipment is available and there are limited amounts of non-fixed equipment | Fixed and non-fixed recess equipment is available to support multiple games and activities | .896 |
| 5 | Hardly any transitions to recess from classroom are organized and smooth | Few transitions to recess from classroom are organized and smooth | Most transitions to recess from classroom are organized and smooth | All transitions to recess from classroom are organized and smooth | .760 |
| 6 | Hardly any supervising adults arrive on time and there are periods of time in which there is no adult supervision | A few supervising adults arrive on time, but supervision is compromised because not enough adults are on the  playground | Most of the supervising adults arrive on time, but a few come out late | All supervising adults arrive on time and there are no periods of time in which students are unsupervised | .695 |

| **1** | | **2** | **3** | **4** | **Kappa (w)** |
| --- | --- | --- | --- | --- | --- |
| 7 | The adult to student ratio is more than 75:1 | The adult to student ratio is between 51-74:1 | The adult to student ratio is approximately 35-50:1 | The adult to student ratio is less than 35:1 | 1.00 |
| 8 | Hardly any organized games and/or activities are available during recess | A limited number of organized games and/or activities are available during recess but there is limited variety | A limited number of organized games and/or activities are available during recess, but there is variety | A variety of organized games and/or activities are available during recess | .791 |
| 9 | Almost all games are exclusive to certain groups by gender, ability, race and/or age (if appropriate) | Some of the games are exclusive to certain groups by gender, ability, race and/or age (if appropriate) | Some of the games are inclusive to certain groups by gender, ability, race and/or age (if appropriate) | Almost all games are inclusive to a variety of groups by gender, ability, race and/or age (if appropriate) | .328 |
| 10 | Hardly any games are initiated by students | A few games are initiated by students | Some games are initiated by students | Almost all games are initiated by students | .782 |
| 11 | Hardly any games are sustained by students | A few games are sustained by students | Some games are sustained by students | Almost all games are sustained by students | .743 |
| 12 | Students have no free choice of activities to play during recess | Students have limited free choice of activities to play during recess | Students have some free choice of activities to play during recess | Students are free to choose the activities to play during recess | .782 |
| 13 | Hardly any students are involved in physically active play | Few students are involved in physically active play | Some students are involved in physically active play | Almost all students are involved in physically active play | .538 |
| 14 | Hardly any of the equipment provided is being used as intended and in a safe manner | Some of the equipment provided is being used appropriately but there are many instances of inappropriate use | Most of the equipment provided is being used appropriately but there are a few instances of inappropriate use | Almost all of the equipment provided is being used as intended and in a safe manner | .507 |

| **1** | | **2** | **3** | **4** | **Kappa (w)** |
| --- | --- | --- | --- | --- | --- |
| 15 | There were several physical altercations between students | There were some physical altercations between students | There were few physical altercations between students | There were no physical altercations between students | .722 |
|  | If **no**, skip the following question and **enter “n/a”** into scoring for the following indicator. | | |  |  |
|  | If **yes**, answer the following question and enter the appropriate score. | |  |  |  |
| 16 | Adults did not intervene between students after seeing physical altercations | Adults intervened after seeing physical altercations but did so in a nonconstructive manner | Adults intervened after seeing physical altercations and sometimes did so in a constructive manner | Adults intervened after seeing physical altercations and almost always did so in a constructive way | NA |
| 17 | Hardly any communication (verbal or nonverbal) between students is positive and encouraging towards each other | Very little communication (verbal or nonverbal) between students is positive and encouraging towards each other | Most of the communication (verbal or nonverbal) between students is positive and is encouraging towards each other | Almost all of communication (verbal or nonverbal) between students is positive and encouraging towards each other | .846 |
|  | Did you observe students using negative verbal or nonverbal communication to each other that was seen by supervising adults? | | | |  |
|  | If **no**, skip the following question and enter “n/a” into scoring for the following indicator. | | |  |  |
|  | If **yes**, answer the following question and enter the appropriate score. | |  |  |  |
| 18 | Adults did not intervene between students after seeing negative communication (verbal or nonverbal) | Adults intervened after seeing negative communication (verbal or nonverbal) but did so in a nonconstructive manner | Adults intervened after seeing negative communication (verbal or nonverbal) and sometimes did so in a constructive manner | Adults intervened after seeing negative communication (verbal or nonverbal) and almost always did so in a constructive way | NA |
| 19 | There were several disagreements about rules between students | There were some disagreements about rules between students | There were few disagreements about rules between students | There were no disagreements about rules between students | .492 |

| **1** | | **2** | **3** | **4** | **Kappa (w)** |
| --- | --- | --- | --- | --- | --- |
| 20 | Students demonstrate hardly any strategies for resolving conflicts on their own | Students demonstrate a few strategies for resolving conflicts on their own, but a lot of adult support was needed | Students demonstrate adequate strategies for resolving conflicts on their own, but some adult support was needed | Students demonstrate strategies to resolve their conflict without adult intervention or there was no evident conflict on the playground | .709 |
| 21 | Hardly any adults model positive culture (e.g. positive language, getting students involved, supporting conflict resolution skills, etc.) | A few adults model positive culture (e.g. positive language, getting students involved, supporting conflict resolution skills, etc.) | Many adults model positive culture (e.g. positive language, getting students involved, supporting conflict resolution skills, etc.) | Almost all adults model positive culture (e.g. positive language, getting students involved, supporting conflict resolution skills, etc.) | .673 |
| 22 | Hardly any of the supervising adults are strategically positioned to view students in the recess play space (i.e., adults are all huddled together) | Some of the supervising adults are strategically positioned to view students in the recess play space, but many students are unsupervised | Many of the supervising adults are strategically positioned to view students in the recess play space, but some students are unsupervised | Almost all of the supervising adults are strategically positioned to view students in the recess play space | .735 |
| 23 | Hardly any adults are playing games or engaged with students | A few adults are playing games and/or are engaged with students | Some adults are playing games and/or are engaged with students | Almost all adults are playing games and engaged with students | .539 |
| 24 | Hardly any transitions to the classroom from recess are organized and smooth | Some transitions to the classroom from recess are organized and smooth | Most transitions to the classroom from recess are organized and smooth | All transitions to the classroom from recess are organized and smooth | .689 |
